# Confirming Rupture of Membranes with Intra-Amniotic Fluorescein Dye Test

**DOI:** 10.1055/a-2724-5458

**Published:** 2025-11-11

**Authors:** Katherine Freedy, Sarah K. Dotters-Katz, Bobby May, Sloane Mebane, Virginia Watkins, Matthew R. Grace, Jennifer Gilner

**Affiliations:** 1 Duke University School of Medicine, Durham, North Carolina, United States; 2 Duke University Department of Obstetrics & Gynecology, Durham, North Carolina, United States; 3 Vanderbilt University Medical Center, Nashville, Tennessee, United States

**Keywords:** PPROM, dye test, fluorescein, tampon

## Abstract

**Introduction:**

We describe two patients presenting with preterm loss of fluid and inconclusive evaluations requiring further assessment. Patient 1 was a 41-year-old G3P1011 at 21 ^6/7^ weeks; Patient 2 was a 22-year-old G2P1001 at 31 ^5/7^ weeks. In both, preterm prelabor rupture of membranes (PPROM) workups yielded mixed results, prompting intra-amniotic dye testing. Due to a national indigo carmine shortage, sodium fluorescein was used. We present photographs of tampons examined under UV light, confirming PPROM in both cases.

**Methods:**

Under ultrasound guidance, 5 cc of sodium fluorescein was injected into the amniotic cavity. Patients wore a tampon for 15 minutes while ambulating. Both provided consent for publication.

**Results:**

On direct visualization, tampons appeared normal. Under UV light (Wood's lamp), the fluorescein emitted a bright neon green fluorescence. Both specimens demonstrated photoluminescence, confirming PPROM.

**Conclusion:**

Although the use of 2 to 5 cc of fluorescein for intra-amniotic dye testing is described in the literature, visual documentation of positive results is limited. These images may guide clinicians in confirming PPROM when indigo carmine is unavailable, supporting fluorescein as a viable diagnostic alternative.

**Key Points:**

- Fluorescein dye can be used to confirm PPROM when tampons are examined under UV light.
- We share unique images of positive fluorescein dye tests to confirm PPROM diagnosis.
- Images may aid diagnosis when indigo carmine is unavailable and fluorescein is used.

## Introduction

The diagnosis of membrane rupture is typically made using nitrazine paper, visualization of fluid within the vagina or “pooling” on speculum exam, and examination of vaginal fluid for ferning under a microscope. The preterm period is arguably the most imperative time frame to confirm preterm prelabor rupture of membranes (PPROM), as it increases the risk of intra-amniotic infection (IAI), preterm labor, and neonatal sepsis. 1 2 In cases where the diagnosis is unclear based on an inconclusive work-up, the instillation of intra-amniotic dye can be used for confirmation of membrane rupture. This test is usually performed with indigo carmine, but a shortage of this drug has led to the identification of alternatives. 3 Reference images of results are lacking in those cases. 4 We present two cases of confirmed PPROM using fluorescein dye.

### Patient 1

Patient 1 is a 41-year-old G3P1011 female, presented to a regional hospital at 21 ^6/7^ weeks with loss of fluid and persistent leaking. Her pregnancy was otherwise complicated by GERD, anxiety/depression, asthma, AMA, and two first-degree relatives with malignant hyperthermia. At presentation, she denied fevers, chills, contractions, or vaginal bleeding. Her vital signs were unremarkable.

Nitrazine test and amnisure were positive, so betamethasone and antibiotics were administered for PPROM, and the patient was transferred to a tertiary care facility. Upon arrival, she began experiencing vaginal bleeding. Speculum exam was notable for bright red blood with no clear source, cervix was closed, and a nonbleeding cervical polyp was observed. There was no ferning visualized on microscopic examination. Interpretation of a positive nitrazine test was confounded in the setting of bleeding. Ultrasound showed amniotic fluid pockets smaller than expected for gestational age (2.1 × 2.4 and 5.7 × 2.6 cm), but no amniotic fluid leak was confirmed. The patient was admitted for presumed PPROM, continued ampicillin and azithromycin, given a second dose of betamethasone, and given magnesium, anticipating a prolonged admission to the antepartum service. Given the discordant findings since transferring from the outside hospital and gestational age, instillation of intra-amniotic dye was recommended.

### Patient 2

The patient, a 22-year-old G2P1001 female, presented to a regional hospital at 31 ^5/7^ weeks with loss of fluid without persistent leaking. Her pregnancy was otherwise complicated by a prior cesarean delivery. At presentation, she denied fevers or chills and endorsed mild lower back pain. Her vital signs were unremarkable.

Her speculum exam was negative for pooling, but microscopic examination of her vaginal swab demonstrated “copious ferning.” Nitrazine paper was unavailable. The patient was admitted with PPROM, started on ampicillin and azithromycin, given betamethasone given magnesium, and transferred to a tertiary center, anticipating a prolonged admission. On arrival, she lacked evidence of labor or intra-amniotic IAI and continued to deny any vaginal leakage. Subsequent ultrasound revealed a normal AFI of 10 cm, and an additional sterile speculum exam was negative the following day. Given discordant exams and the patient's desire to leave the hospital, the patient opted for the instillation of intra-amniotic dye.

## Methods

**Fig. 1:**
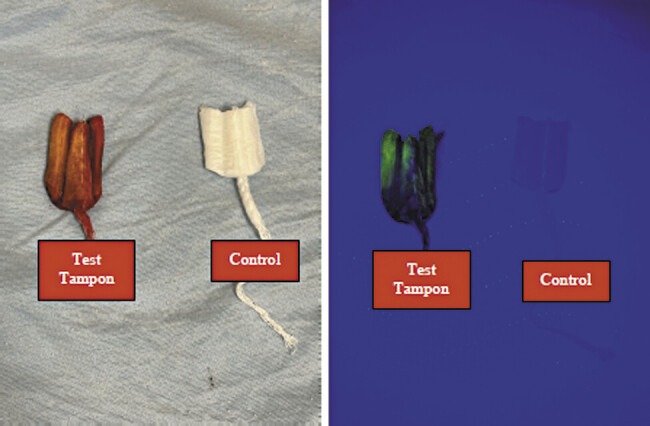
Tampons by naked eye (right) and under fluorescent light (left), dye test tampon on right, unused tampon on right from patient case 1.

**Fig. 2:**
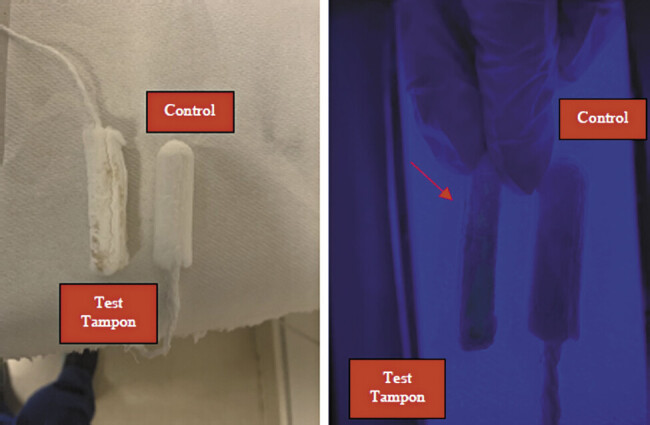
Tampons by naked eye (right) and under fluorescent light (left), dye test tampon on right, unused tampon on right from patient case 2.

In both cases, due to a shortage of indigo carmine, 5 cc of sodium fluorescein was injected into the amniotic cavity under ultrasound guidance. The patients were instructed to wear a tampon for 30 minutes while ambulating. The tampon appeared normal on direct visualization ( Figs. 1 and 2 ). However, under UV light, the tampon exhibited photoluminescence, confirming the presence of fluorescein and the diagnosis of PPROM ( Figs. 1 and 2 ).

## Discussion

### Patient 1

The patient remained on the antepartum service until hospital day 49, at which point she developed contractions, abdominal pain, and malaise, concerning for IAI. She underwent cesarean delivery at 28 ^6/7^ weeks and was treated with ampicillin and gentamycin. Ultimately, the patient's postpartum course was uncomplicated, and she was discharged postpartum day 3. The neonate was admitted to the intensive care nursery (ICN) and was discharged home after 67 days.

### Patient 2

The patient remained on the antepartum service until hospital day 5, at which point she developed fevers and fetal tachycardia, concerning for IAI. She underwent repeat cesarean delivery and was treated for presumed endometritis due to continued fevers after delivery. Ultimately, the patient did well and was discharged postpartum day 3. The neonate was admitted to the ICN and was discharged home after 48 days.

## Conclusion

In an intraamniotic indigo-carmine dye test, the tampon is visibly blue. In contrast, fluorescein requires the use of a UV light (in our case, a Wood's lamp) and displays a bright yellow appearance ( Fig. 2 ). Though the technique is well described in the literature, images of positive results are limited. These images may provide guidance in confirming PPROM diagnoses in similar cases.

## References

[1] Hoffman M K Prediction and prevention of spontaneous preterm birth: ACOG practice bulletin, number 234 Obstet Gynecol 2021 138 06 945 946 10.1097/AOG.0000000000004612 PMC8607754 34794160

[2] Committee Opinion No Committee opinion no. 797: prevention of group B streptococcal early-onset disease in newborns: correction Obstet Gynecol 2020 135 04 978 979 10.1097/AOG.0000000000003824 32217968

[3] American College of Obstetricians and Gynecologists' Committee on Practice Bulletins—Obstetrics Practice bulletin no. 172: premature rupture of membranes Obstet Gynecol 2016 128 04 e165 e177 27661655 10.1097/AOG.0000000000001712

[4] Ireland K E Rodriguez E I Acosta O M Ramsey P S Intra-amniotic dye alternatives for the diagnosis of preterm prelabor rupture of membranes Obstet Gynecol 2017 129 06 1040 1045 28486367 10.1097/AOG.0000000000002056

